# Toxicity of High-Density Polyethylene Nanoparticles in Combination with Silver Nanoparticles to Caco-2 and HT29MTX Cells Growing in 2D or 3D Culture

**DOI:** 10.3390/molecules31010003

**Published:** 2025-12-19

**Authors:** Sylwia Męczyńska-Wielgosz, Katarzyna Sikorska, Malwina Czerwińska, Agnieszka Grzelak, Anna Lankoff, Marcin Kruszewski

**Affiliations:** 1 Centre for Radiobiology and Biological Dosimetry, Institute of Nuclear Chemistry and Technology, 03-195 Warsaw, Poland; s.meczynska@ichtj.waw.pl (S.M.-W.); k.sikorska@ichtj.waw.pl (K.S.); m.czerwinska@ichtj.waw.pl (M.C.); m.kruszewski@ichtj.waw.pl (M.K.); 2 Centre for Digital Biology and Biomedical Science—Biobank Łódź, Faculty of Biology and Environmental Protection, University of Lodz, 91-237 Lodz, Poland; agnieszka.grzelak@biol.uni.lodz.pl; 3 Department of Medical Biology, Institute of Biology, Jan Kochanowski University, Uniwersytecka 7, 25-406 Kielce, Poland; 4 Department of Molecular Biology and Translational Research, Institute of Rural Health, 20-090 Lublin, Poland

**Keywords:** nanoplastic, HDPE nanoparticles, silver nanoparticles, cellular uptake, toxicity, 2D monoculture, 3D triple-culture

## Abstract

The enormous applications of various nanoparticles (NPs) have raised the possibility that humans may be simultaneously exposed to mixtures of them in real life. Realistically, this situation may apply to plastic NPs, mainly derived from the breakdown of larger plastics, and to silver NPs, both of which are among the most frequently detected NPs in the envirnment due to their applications in healthcare, consumer products, and water purification. Although numerous studies have examined the toxicity of plastic and silver NPs individually, knowledge of their combined toxicity remains limited. Hence, the main objective of our study was to investigate the toxicity of high-density polyethene nanoparticles (HDPE NPs), thermally isolated from food-cooking bags, in combination with citrate-stabilised silver nanoparticles (AgNPcit) to Caco-2 and HT29MTX cells growing in 2D monoculture or in 3D triple-culture with Raji cells. Cellular uptake of NPs was quantified from the side-scatter (SSC) signal in flow cytometry; toxicity was evaluated by the neutral red assay; apoptosis was evaluated by the Annexin V method; and induction of oxidative stress was evaluated by a fluorescent method using DCFDA and DHR probes. Both cell lines took up both types of NPs; however, HT29MTX cells were more effective in the NPs’ uptake. Interestingly, HDPE NPs and AgNPcit mutually inhibited each other’s uptake, which suggests a similar mechanism of entry. Both types of NPs were toxic to both cell lines growing in monoculture; Caco-2 cells were more susceptible than HT29MTX. The toxicity was attributed to the induction of oxidative stress and associated apoptosis. In line with the mutual inhibition of the NPs’ uptake, the toxic effect of both NPs in the mixture was less than that expected as the sum of individual treatments. The toxic effects of both NPs or their mixture were less pronounced in the triple-culture Caco-2/HT29MTX/Raji, than in Caco-2 and HT29MTX growing in monoculture.

## 1. Introduction

The enormous applications of various NPs in industry, agriculture, medicine, environment, electronics, energy and consumer goods [1,2] have raised the possibility that humans may be simultaneously exposed to mixtures of them in real life. Indeed, many studies confirm that humans and the environment are constantly exposed to complex nanoparticle mixtures rather than to individual types of particles [3,4,5,6,7,8,9]. As a result, growing concerns focus on the interactive and cumulative effects of nanoparticle mixtures on human health. While there is extensive evidence on the toxicity of single particle types, a significant knowledge gap exists regarding the toxic effects arising from their combined exposure. In response to limited knowledge of the harmful effects of combined exposure to nanoparticles, the current study was generally designed to investigate the toxicity of plastic NPs, derived from the breakdown of human-made plastics through natural processes, in combination with silver NPs, both of which are among the most frequently detected NPs in the environment [10,11].

Plastic NPs and silver NPs may enter the human body by three major exposure pathways: inhalation, ingestion, and skin contact. Skin forms a protective barrier against the external environment; therefore, direct uptake of plastic NPs and silver NPs through the skin is not expected, unless they are intentionally applied as components of cosmetics, wound dressings, etc. The lungs, due to their large alveolar surface area, are generally considered an important route of exposure to various nanoparticles. However, the concentration of plastic NPs and silver NPs in the air is usually low. Therefore, ingestion appears to be one of the primary routes by which they enter the human body. A range of studies reported that plastic NPs can pass through the gut epithelium and enter the body [12]. Moreover, the available data provided evidence for the toxic potential of plastic NPs and silver NPs in the gut epithelium. A recent review by Yong et al. [13] highlighted multiple pathological processes following exposure to plastic NPs, including gut dysbiosis, intestinal barrier dysfunction, metabolic changes, increased oxidative stress, and signs of neurotoxicity. Additionally, in vitro experiments on various human cell lines showed that plastic NPs elicit oxidative stress, an inflammatory response, and cytotoxicity [13,14]. Similarly, the existing literature clearly indicated that silver NPs have toxic effects on intestinal permeability in an in vitro model of the human gut epithelium [15]. In vivo investigations also confirmed the adverse impact of silver NPs on the gut, including damage to the epithelial structure, reduced thickness of the mucosal layer, and alterations in the intestinal immune microenvironment [16], gut microbiota, and metabolic profile [17]. Once plastic NPs and silver NPs enter the gut, they may overcome primary tissue barriers and may be transported through the bloodstream to secondary organs. Grafmueller et al. [18] showed that polystyrene particles with diameters of up to 240 nm can be taken up by cells of the placental barrier and transported from the foetal to the maternal blood circulation.

One of the factors restricting our knowledge about the health impact of plastic NPs is the limited number of studies on environmentally originated plastic NPs; thus, the majority of our current knowledge regarding their interaction with the body comes from cell culture studies using commercially produced polystyrene NPs, created for scientific research as model plastic NPs. Forte et al. [19] demonstrated that polystyrene nanoparticles with a diameter of 44 nm accumulate more rapidly and efficiently in the cytoplasm of human gastric adenocarcinoma cells than similar 100 nm particles. Walczak et al. [20,21] showed that the uptake of polystyrene particles with diameters of 50 and 100 nm by three intestine cell lines, Caco-2, HT29MTX, and 63 M-cells, strongly depended on the particles’ surface charge and size. In contrast, 1, 4, and 10 μm polystyrene particles were hardly taken up by the human intestinal epithelial cell line Caco-2 and its co-cultures, which mimic intestinal M-cells and goblet cells [22]. More recently, we confirmed poor internalisation of polystyrene nanoparticles (PS NPs) by Caco-2 cells, suggesting receptor-mediated endocytosis as the primary mechanism of internalisation. Furthermore, our study confirmed the low toxicity of PS NPs despite their apparent cellular uptake [23]. The reliance on model polystyrene NPs is a significant limitation because they are usually homogeneous in size and shape and may not reflect the behaviour of plastic NPs originating from the natural degradation of larger plastic waste in real-world processes. While studies on model polystyrene NPs help us understand mechanisms of their interactions with living organisms, assigning them directly to environmental conditions is problematic, as man-made model plastic NPs do not resemble naturally weathered plastic NPs present in environmental matrices. This is mainly because naturally weathered plastic NPs have various sizes, shapes and different surface modifications.

Thus, to fill the gap in our knowledge of the health impact of naturally weathered plastic NPs, the specific objective of this research was to investigate the toxicity of thermally isolated high-density polyethene nanoparticles (HDPE NPs) in combination with citrate-stabilised silver nanoparticles (AgNPcit) to Caco-2 and HT29MTX cells growing in 2D monoculture or in 3D triple-culture with Raji cells. In this work, we used HDPE NPs prepared from a bulk polyethene material (plastic bags used for cooking rice, grains, etc.) through thermal, natural degradation, which is very likely to occur in a household during daily activities. To test the NPs’ physical properties and nature of their colloidal interactions, a series of experiments were performed using high-resolution scanning electron microscopy (HR-SEM), dynamic light scattering (DLS), and nanoparticle tracking analysis (NTA). Cellular uptake of HDPE NPs and AgNPcit by two human cell lines growing in 2D monoculture (human colorectal adenocarcinomas: Caco-2 and mucus-secreting, goblet-like HT29MTX) was quantified from the side-scatter (SSC) signal in flow cytometry. Toxicological investigations of HDPE NPs and AgNPcit were performed using the neutral red assay (toxicity), the Annexin V method (apoptosis), and a fluorescent method using DCFDA and DHR probes (induction of oxidative stress) in a 3D triple culture (Caco-2/HT29MTX/Raji). The Caco-2/HT29MTX cells were seeded at a 70:30 ratio to mimic the proportions of epithelial and goblet cells in the large intestine, or at a 90:10 ratio to mimic the proportions in the small intestine.

## 2. Results

### 2.1. Characterisation of HDPE Particles

**Figure 1:**
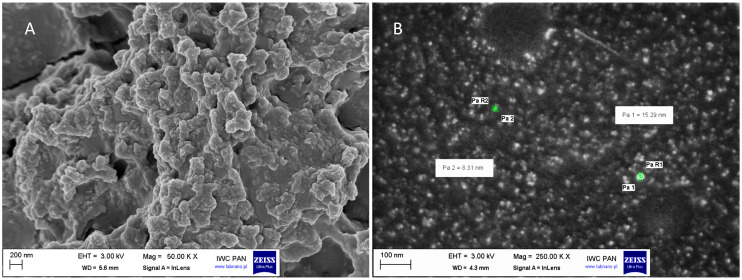
Scanning electron microscopy (SEM) images of high-density polyethylene nanoparticles (HDPE NPs) at different magnifications. (**A**) Detail of the HDPE NPs agglomerate at 50,000× magnification, scale bar 200 nm, and (**B**) detail of the HDPE NPs at 250,000× magnification, scale bar 100 nm. The observations were performed in InLens mode at an accelerating voltage of 3.00 kV. The isolated HDPE NPs, suspended in deionised water and not coated, were dropped into aluminium specimen stubs and sprayed with a thin layer of carbon.

The morphology, shape, and size distribution of HDPE nanoparticles were examined using scanning electron microscopy (SEM), as presented in Figure 1.

The micrograph 1A clearly shows that the particles exhibit a pronounced tendency to form large, irregularly shaped aggregates. These agglomerates are composed of multiple fused or clustered particles, which vary in size and are often intertwined in complex, non-spherical forms. The average size of the aggregated structures ranges from 100 to 200 nm. Further inspection of the surface morphology revealed that the majority of the HDPE NPs exhibited a rough, irregular shape (Figure 1B). SEM analysis confirms that the HDPE particles isolated from cooking bags are morphologically diverse, with a clear propensity for aggregation and structural irregularity, which may affect their physicochemical behaviour and stability in suspension.

The results of Nanoparticle Tracking Analysis (NTA) analysis and Dynamic Light Scattering (DLS) measurements are provided below in Table 1, Table 2, Table 3 and Table 4. Nanoparticle Tracking Analysis (NTA) analysis: Table 1 and Table 3; Dynamic Light Scattering (DLS) measurements: Table 2 and Table 4.

The hydrodynamic diameter of isolated HDPE particles, measured by NTA, was markedly smaller than the size determined by DLS. The hydrodynamic diameter determined by NTA at time 0 in PBS, HT29 cells growth medium, or Caco-2 cells growth medium was 198.7 ± 23.5 nm, 207.0 ± 20.1 nm, and 209.3 ± 25.4 nm, respectively. After 24 h, the particle size increased to 239 ± 30.4 nm, 243 ± 29.4 nm, and 245 ± 38.7 nm, respectively, reflecting the temporal kinetics of particle size evolution. However, in contrast, DLS measurements revealed significantly larger hydrodynamic diameters, with values of 245.23 ± 1.23 nm, 260.66 ± 0.77 nm, and 265.20 ± 1.32 nm for time 0 in PBS, HT29 cell, and Caco-2 cell growth media, respectively. After 24 h, the DLS measurements revealed a further increase in the diameter of HDPE NPs (up to 299.31 ± 2.98 nm, 352.43 ± 4.33 nm, and 361.82 ± 1.02 nm in the respective media).

In addition to estimating the particle size, NTA was also used to estimate particle number concentration (particles/mL) over time. The concentration of microplastic particles in all tested media declined progressively over time, confirming time-dependent aggregation behaviour. In phosphate-buffered saline (PBS), the initial concentration of 5.0 × 10^9^ ± 0.59 particles/mL decreased to 2.95 × 10^9^ ± 0.47 particles/mL after 24 h. A similar trend was observed in both HT-29 cell and Caco-2 cell culture media, where the particle concentrations declined from 4.87 × 10^9^ ± 0.47 to 3.14 × 10^9^ ± 0.43 particles/mL, and from 5.30 × 10^9^ ± 0.63 to 3.23 × 10^9^ ± 0.51 particles/mL, respectively.

The surface charge of HDPE particles, determined using the DLS method, revealed high negative values of zeta potential, measured as follows: −37.91 ± 1.55 mV in PBS, −35.16 ± 5.21 mV in HT29 medium, and −35.63 ± 2.03 mV in Caco-2 medium at time 0. Additionally, a kinetic analysis of zeta potential changes over time was performed by measuring the surface charge at multiple time points (Table 2). After 24 h, the zeta potential values reached approximately −30 mV in all examined media (PBS, HT29 cell medium, and Caco-2 cell medium).

The polydispersity index (PDI) values for HDPE particles were 0.422 ± 0.026 for PBS, 0.488 ± 0.032 for HT29 medium, and 0.486 ± 0.030 for Caco-2 medium, respectively, at time 0, indicating a broad (polydisperse) particle size distribution. After 24 h, these values increased to 0.732 ± 0.061, 0.821 ± 0.071, and 0.907 ± 0.090, respectively, suggesting a further broadening of the size distribution over time. The numerical value of the PDI ranges from 0 (representing a perfectly monodisperse sample with uniform particle size) to 1.0 (indicating a highly polydisperse system with a wide distribution of particle sizes). An increase in PDI over time may result from processes such as aggregation or adsorption of biomolecules onto the particle surface, leading to greater heterogeneity in particle size.

### 2.2. Characterisation of Ag Particles

Measurements of the hydrodynamic diameter of AgNPcit by NTA and DLS revealed similar regularities to those of HDPE NPs. DLS measurements resulted in a much higher hydrodynamic diameter than NTA measurements; however lower polydispersity index of AgNPcit dispersion suggested better homogeneity of the size distribution. The hydrodynamic diameter of AgNPcit measured in the culture media was higher than that measured in PBS. Both methods indicated a tendency for the AgNPcit to agglomerate. The initial hydrodynamic diameter of AgNPcit measured in PBS by NTA was estimated to be 36.2 ± 3.1 nm, and was slightly higher than declared by the manufacturer (20 nm). Zeta potential of AgNPcit was similar to that measured for HDPE NPs (approx. −35 mV).

### 2.3. Endotoxin Content

Endotoxin content was estimated as 162.2 EU/μg/mL (one experiment only) for HDPE NPs and 74.1 EU/μg/mL (range 42.6–105.6) for AgNPcit, roughly corresponding to 32.4 ng/μg/mL and 14.8 ng/μg/mL endotoxin, respectively.

### 2.4. Nanoparticle Uptake by Caco-2 or HT29MTX Cells Growing in Monoculture

**Figure 2:**
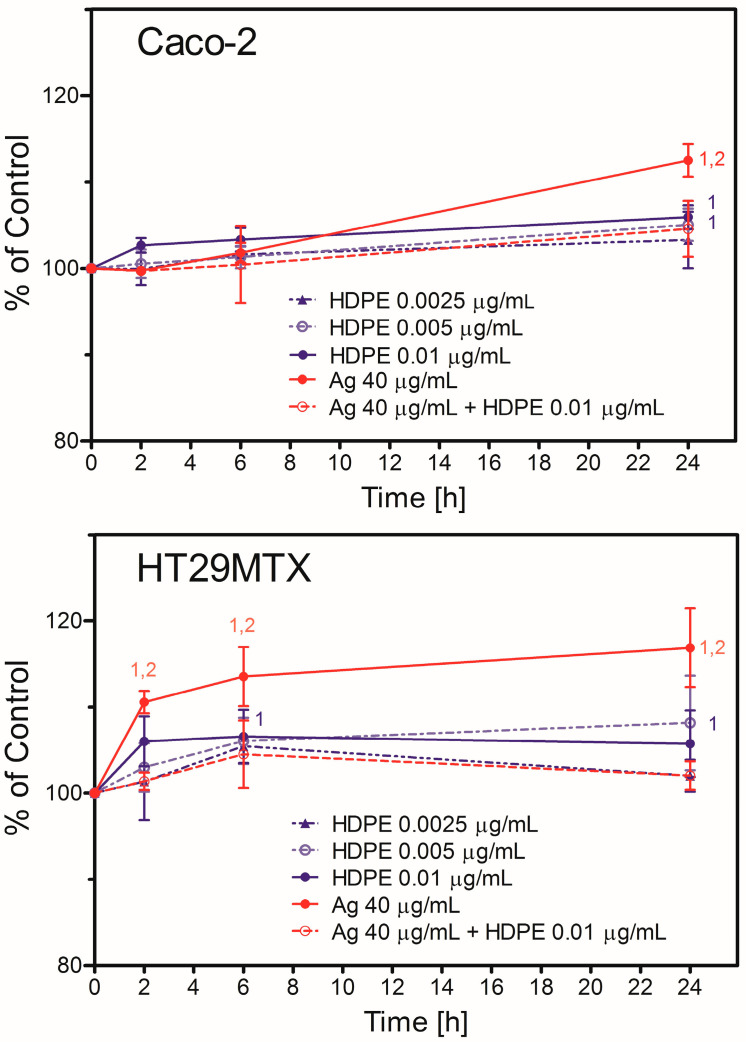
Uptake of AgNPcit and HDPE NPs by Caco-2 or HT29MTX cells growing in monoculture (2D model). HDPE NPs—high-density polyethene nanoparticles; Ag—silver nanoparticles. (1) denotes statistically significant difference from the Control, *p <* 0.05, (2) denotes statistically significant difference from HDPE NPs + AgNPcit, *p <* 0.05. Data are presented as Mean ± SD from 3 independent experiments. Statistical analysis was performed on the raw data. For HDPE NPs, a two-way repeated-measures ANOVA followed by a Bonferroni post hoc test was used; otherwise, a one-way repeated-measures ANOVA followed by a Tukey post hoc test was used.

Caco-2 and HT29MTX cells differed regarding nanoparticle uptake. HT29MTX cells took up both kinds of NPs more readily than Caco-2 cells (Figure 2). 

For HT29MTX cells, uptake of AgNPcit was statistically significant as soon as 2 h after the addition of NPs and continued to grow up to 24 h (the longest time tested). The uptake of HDPE NPs in the highest concentration used was also significant 6 h after the addition of NPs and remained at the same level up to 24 h. Although the uptake of HDPE NPs in low concentrations was also observed, it never reached statistical significance, except at a concentration of 0.005 μg/mL after 24 h of treatment. In contrast, uptake of both kinds of NPs by Caco-2 cells was much slower, and statistical significance was observed only 24 h after the NPs’ addition for AgNPcit, HDPE NPs at concentrations 0.01 μg/mL and 0.005 μg/mL. Interestingly, in both cell lines, the presence of HDPE NPs inhibited the uptake of AgNPcit, as the uptake of both NPs in a mixture was significantly lower than that of AgNPcit alone (Figure 2).

### 2.5. Nanoparticle Toxicity in Caco-2 or HT29MTX Cells Growing in Monoculture

**Figure 3:**
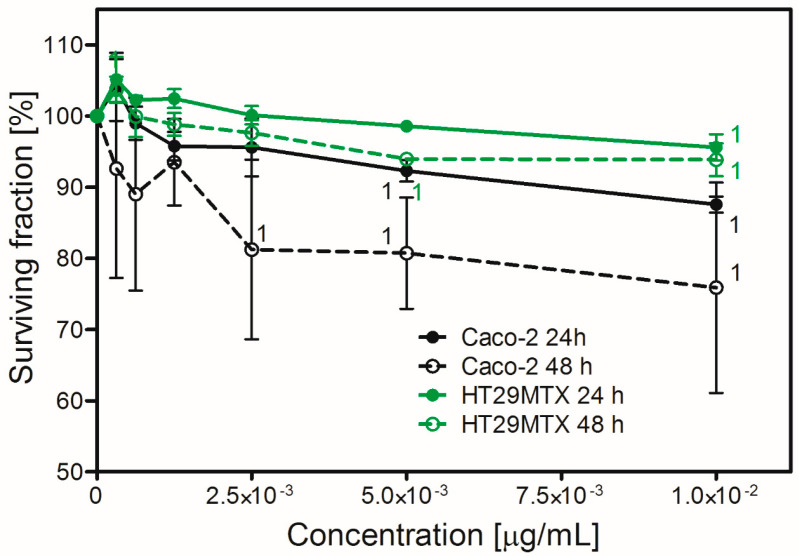
Toxicity of HDPE NPs to Caco-2 or HT29MTX cells growing in monoculture (2D model). HDPE NPs—high-density polyethene nanoparticles. (1) denotes a statistically significant difference from the control. *p* < 0.05. Data are presented as Mean ± SD from 3 independent experiments. Statistical analysis was performed on the raw data. Data from the individual experimental groups were analysed using One-way ANOVA followed by Dunnett post hoc comparison.

The toxicity of HDPE NPs is shown in Figure 3. HT29MTX cells did not exhibit a significant decrease in survival upon treatment with HDPE NPs. After 24-h treatment slight induction of proliferation was visible but did not reach statistical significance. After 48 h of treatment, a small reduction in survival was observed for the two highest concentrations tested. Although statistical analysis revealed a significant result, the survival rate was still approximately 95% of that of the control. In contrast, Caco-2 cells were more vulnerable to the treatment with HDPE NPs. A statistically significant decrease in survival was observed after 24-h treatment for the two highest concentrations tested and for the three highest concentrations tested after 48-h treatment.

**Figure 4:**
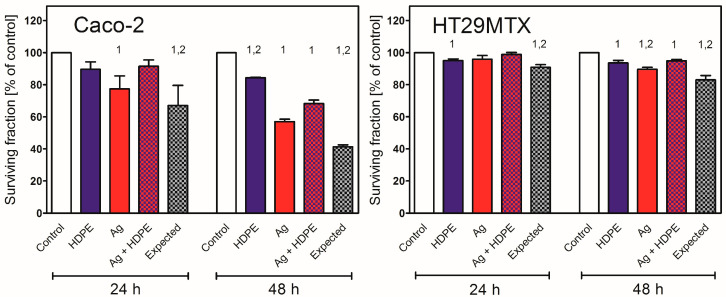
Toxicity of HDPE NPs (0.01 μg/mL), AgNPcit (15 μg/mL), and their mixture to Caco-2 or HT29MTX cells growing in monoculture (2D model). HDPE NPs—high-density polyethene nanoparticles; Ag—silver nanoparticles. (1) denotes a statistically significant difference from Control. (2) denotes a statistically significant difference from the experimental result of the combination of HDPE NPs +AgNPcit. *p* < 0.05. Data are presented as Mean ± SD from 3 independent experiments. Statistical analysis was performed on the raw data. Data from the individual experimental groups were analysed using One-way ANOVA followed by Tukey post hoc comparison.

The toxicity of HDPE NPs was also evaluated in combination with AgNPcit (15 μg/mL). In this concentration, AgNPcit were toxic for both cell lines; however, when used in combination with HDPE, their toxicity was attenuated. Although the toxicity of AgNPcit alone was similar to the toxicity of both NPs used together (except HT29MTX cells treated for 48 h), in all cases the expected value for combined treatment was significantly lower than the actual experimental value (Figure 4 treated for 48 h), in all cases the expected value for combined treatment was significantly lower than the actual experimental value (Figure 4).

### 2.6. Induction of Apoptosis in Caco-2 or HT29MTX Cells Growing in Monoculture or Triple-Culture Caco-2/HT29MTX/Raji

**Figure 5:**
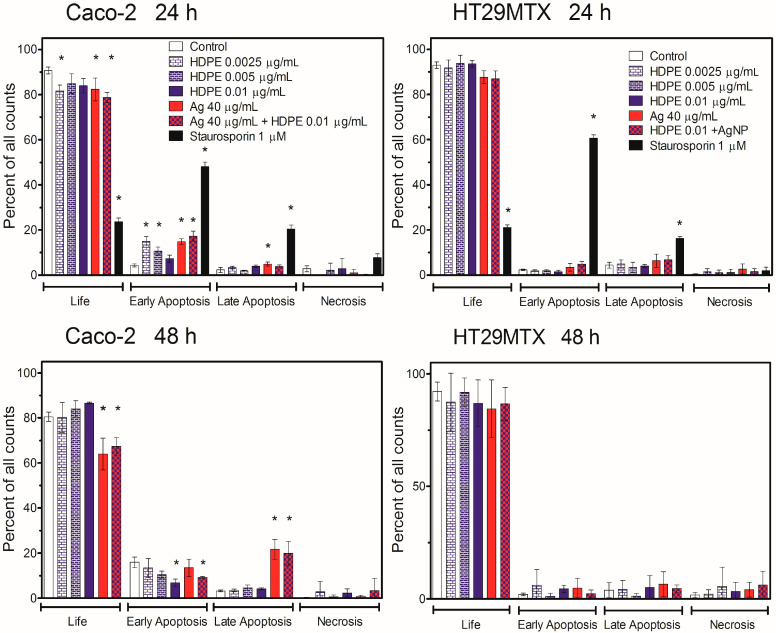
Induction of apoptosis in Caco-2 or HT29MTX cells growing in monoculture (2D model). HDPE NPs—high-density polyethene nanoparticles; Ag—silver nanoparticles; Staurosporin—positive control. Asterix denotes statistically significant difference from control, *p <* 0.05. Data are presented as Mean ± SD from 3 independent experiments. Statistical analysis was performed on the raw data. Data from the individual experimental groups were analysed using One-way ANOVA followed by a Dunnett post hoc test.

**Figure 6:**
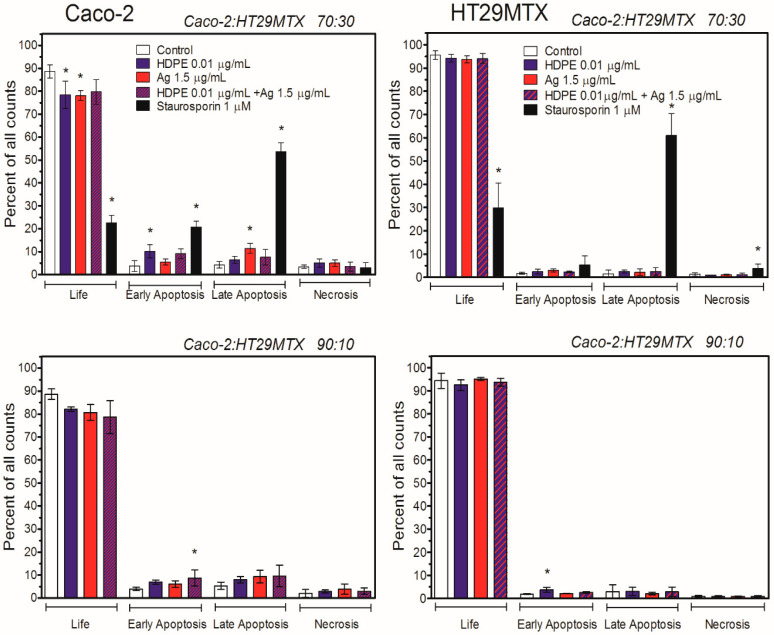
Induction of apoptosis in Caco-2 and HT29MTX cells growing in a 3D triple-culture 24 h after NPs addition. HDPE NPs—high-density polyethene nanoparticles; Ag—silver nanoparticles; Staurosporin—positive control. Asterix denotes statistically significant difference from control, *p* < 0.05. Data are presented as Mean ± SD from 3 independent experiments. Statistical analysis was performed on the raw data. Data from the individual experimental groups were analysed using One-way ANOVA followed by a Dunnett post hoc test.

The induction of apoptosis by HDPE NPs, AgNPcit, or their mixture was tested in Caco-2 and HT29MTX cells growing in monoculture (Figure 5) or growing in triple-culture Caco-2/HT29MTX/Raji (Figure 6).

In the monoculture of Caco-2 cells, a statistically significant increase in the number of cells in the early apoptotic stage was observed for all treatments after 24 h. After 48-h treatment, the number of early apoptotic cells decreased, while the number of late apoptotic cells increased, especially in the case of treatment with AgNPcit and their mixture with HDPE. In contrast, no statistically significant signs of apoptosis were observed in HT29MTX cells (Figure 5). A similar picture appeared in the 3D scheme. In the triple-culture with the Ca-co-2/HT29MTX cells ratio of 70:30, after 24-h treatment, the number of Caco-2 cells in the early apoptotic stage was significantly higher in cells treated with the highest concentration of HDPE NPs, whereas the number of cells in the late apoptotic stage was significantly higher in cells treated with AgNPcit. In the triple-culture with the Ca-co-2/HT29MTX cells ratio of 90:10, the only significant difference observed was an in-crease in the number of early apoptotic cells in cultures treated with the mixture of AgNP-cit + HDPE NPs. In contrast, no statistically significant increase in the number of apoptotic cells was observed in HT29MTX cells (Figure 6) with the exception of the early apoptotic fraction of cells treated with HDPE NPs in the setup with the Caco-2/HT29MTX ratio 90:10.

### 2.7. Oxidative Stress in Caco-2 or HT29MTX Cells Growing in Monoculture or Triple-Culture Caco-2/HT29MTX/Raji

**Figure 7:**
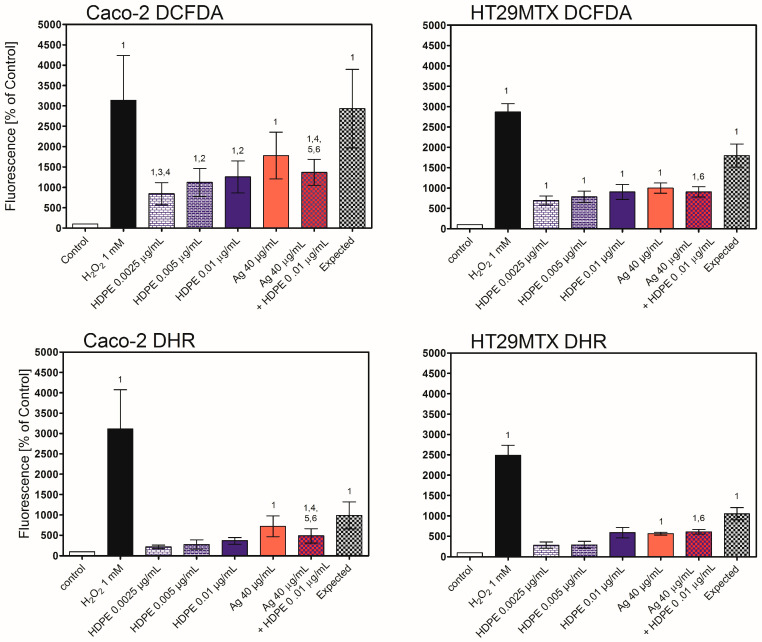
Generation of Reactive Oxygen Species in Caco-2 and HT29MTX cells treated with AgNPcit and HDPE NPs in monoculture culture (2D model). DHR—1,2,3-dihydrorhodamine, DCFDA—2′,7′-dichlorodihydrofluorescein diacetate, HDPE NPs—high-density polyethene nanoparticles; Ag—silver nanoparticles; H_2_O_2_—hydrogen peroxide as positive control. (1) denotes statistically significant difference from control; (2) denotes statistically significant difference from HDPE NPs in concentration 0.0025 μg/mL, *p <* 0.05, (3) denotes statistically significant difference from HDPE NPs in concentration 0.005 μg/mL, *p <* 0.05, (4) denotes statistically significant difference from HDPE NPs in concentration 0.01 μg/mL, *p <* 0.05, (5) denotes statistically significant difference from the mixture of AgNPcit (40 μg/mL) + HDPE NPs (0.01 μg/mL), *p* < 0.05, (6) denotes statistically significant difference from the expected value, *p <* 0.05. Data are presented as Mean ± SD from 3 independent experiments. Statistical analysis was performed on the raw data. Data from the individual experimental groups were analysed using One-way ANOVA followed by the Tukey post hoc test. The concept of expected value is explained in the Materials and Methods section.

Oxidative stress induced by HDPE NPs, AgNPcit, or their mixture was tested in 2D and 3D models using two fluorescent probes, DCFDA and DHR. In 2D model, DCFDA revealed intense production of Reactive Oxygen Species (ROS) in cells treated with HDPE NPs in the whole range of tested concentrations (Figure 7). The DCFDA probe also revealed production of the ROS in AgNPcit-treated cells and in cells treated with a mixture of HDPE NPs and AgNPcit. Interestingly, the ROS production by the cells treated with HDPE NPs + AgNPcit mixture was only slightly lower than by AgNPcit alone, and much lower than might be expected from the additive effect of both NPs. DHR probe indicated a lower level of ROS production, as a statistically significant increase was observed only for the treatment with AgNPcit and the mixture of HDPE NPs + AgNPcit. Nevertheless, similarly to DCFDA, the ROS production by cells treated with HDPE NPs + AgNPcit mixture was only slightly lower than that treated with AgNPcit alone, and lower than might be expected from the additive effect of both NPs (Figure 7).

**Figure 8:**
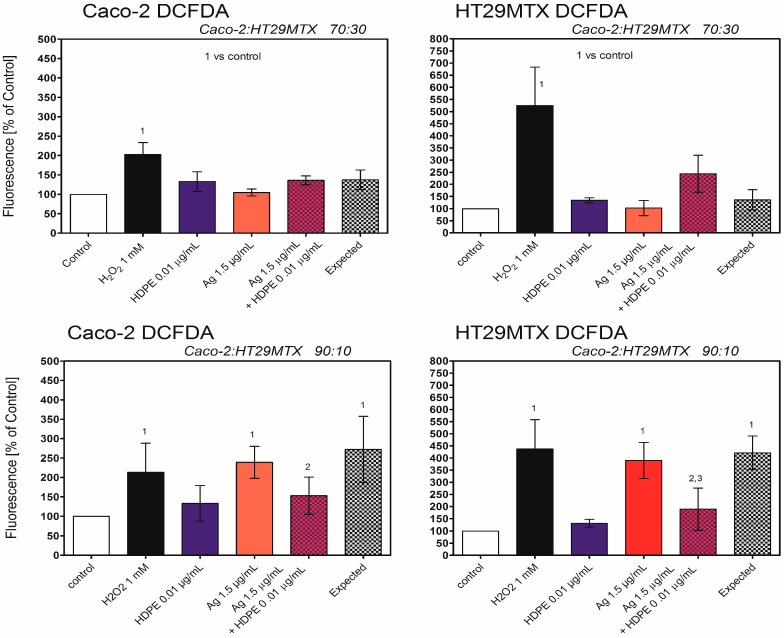
Generation of Reactive Oxygen Species in Caco-2 and HT29MTX cells treated with AgNPcit and HDPE NPs in 3D triple-culture, determined by DCFDA. HDPE NPs—high-density polyethene nanoparticles; Ag—silver nanoparticles; H_2_O_2_—hydrogen peroxide as positive control. (1) denotes statistically significant difference from control, (2) denotes statistically significant difference from expected, (3) denotes statistically significant difference from AgNPcit, *p <* 0.05. Data are presented as Mean ± SD from 3 independent experiments. Statistical analysis was performed on the raw data. Data from the individual experimental groups were analysed using One-way ANOVA followed by the Tukey post hoc test. The concept of expected value is explained in the Materials and Methods section.

**Figure 9:**
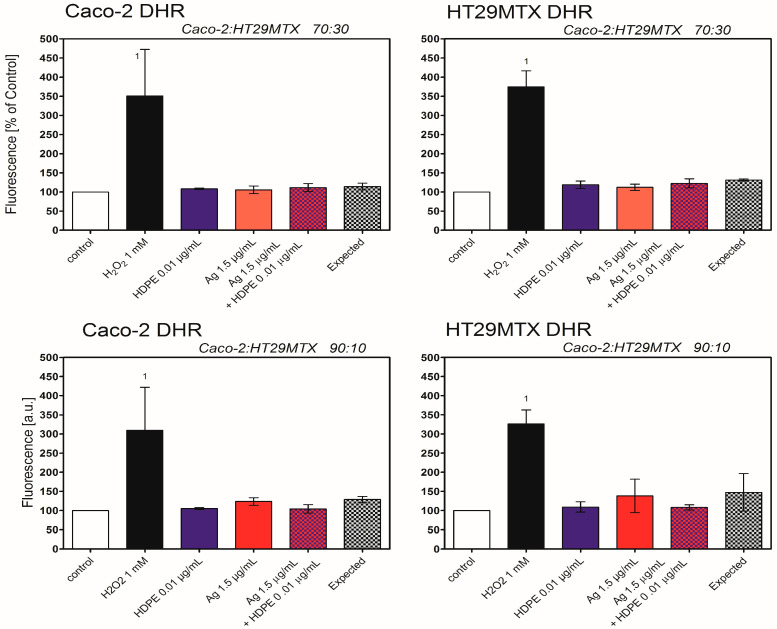
Generation of Reactive Oxygen Species in Caco-2 and HT29MTX cells treated with AgNPcit and HDPE NPs in 3D triple-culture, determined by DHR. DHR—1,2,3-dihydrorhodamine, DCFDA—2′,7′-dichlorodihydrofluorescein diacetate, HDPE NPs—high-density polyethene nanoparticles; Ag—silver nanoparticles; H_2_O_2_—hydrogen peroxide as positive control. (1) denotes statistically significant difference from control, *p* < 0.05. Data are presented as Mean ± SD from 3 independent experiments. Statistical analysis was performed on the raw data. Data from the individual experimental groups were analysed using One-way ANOVA followed by the Tukey post hoc test. The concept of expected value is explained in the Materials and Methods section.

No induction of oxidative stress was observed in 3D triple-culture, regardless the cell line, used fluorescent probe, or proportion of Caco-2/HT29MTX cells, with exception of Caco-2 and HT29MTX cells in ratio 90:10, treated with AgNPcit (40 μg/mL) (Figure 8 and Figure 9).

## 3. Discussion

Recent reports indicate that humans may be simultaneously exposed to mixtures of various nanomaterials in real life. Realistically, this situation may apply to silver NPs and plastic NPs, which are among the most frequently detected NPs in the environment. Hence, human exposure to them is very likely. While the majority of studies on silver NPs indicate their substantial toxicity [24], hopefully, the majority of studies on model plastic nanoparticles, such as polystyrene NPs, indicate low or moderate toxicity [25,26]. However, the toxicity of model NPs, which are usually uniform in size and shape, does not necessarily reflect the actions of NPs that result from natural weathering or in-home processes, such as cooking. Thus, we conducted a study on a mixture of silver NPs and plastic NPs, thermally released from food packaging foil commonly used in everyday life.

Our results revealed that boiling of perforated polyethylene foil, used to produce rice cooking bags, in water at 95–100 °C for 1 h resulted in particle leaching from a bulk material. These results are in line with studies of Deng et al. [27], who applied similar methodology to obtain particles, and showed that high-heat treatment of commercial plastic products (polyethylene terephthalate (PET), polyethylene (PE), low density polyethylene (LDPE) and polypropylene (PP), commonly used for food packaging, leads to the release of microplastics and nanoplastics, which are of various shapes and size. In addition, Liu et al. [28] reported that a 1-h hot-water (100 °C) treatment of disposable plastic materials (plastic packaging, cups, transparent boxes, and expandable boxes) resulted in the release of nano- and microparticles. More recently, findings of Li et al. [29] revealed that feeding bottles, food containers, and paper cups released microplastics and nanoplastics after heating at 70–100 °C

Such in-home-generated NPs might be contaminated with various environmental substances. One of the most abundant substances to which humans are exposed every day is the remains of bacteria, known as lipopolysaccharides, or more generally, endotoxins. Endotoxins are the main components of the outer membrane of the Gram-negative bacterial cell wall. They are typically released after mechanical damage to bacteria or upon their death, as well as during division. If endotoxins enter human or animal blood, they may trigger various biological responses, including fever or septic shock, even at low levels. As Gram-negative bacteria are widespread in the environment, endotoxins are also ubiquitous, often attached to surfaces, dust, and particulate matter. Such prevalence makes exposure to endotoxins unavoidable [30]. Since in this work we used NPs prepared from commercially available row material in non-sterile conditions mimicking everyday activities, their contamination with widespread bacterial endotoxins and pyrogens was very likely. Indeed, bacterial endotoxin content in our NP preparation was relatively high. However, we intended to use a material obtained through a standard in-house procedure, such as cooking, with all the consequences of that process. Nonetheless, in our study, endotoxin concentration in the culture medium with the highest NP concentration (40 μg/mL AgNPcit) used to treat the cells was calculated to be about 592 ng/mL, and likely did not affect Caco-2 cells survivability, as no toxic effect of LPS was observed in these cells till the concentration of 10 μg/mL [31]. This is also true for HDPE NP suspension, where the endotoxin concentration in the solution with the highest NP concentration was calculated to be 0.32 ng/mL.

Physical analysis of the HDPE NPs released from food packaging foil during cooking revealed particles with irregular shapes and sizes, a negative surface potential, and a slight tendency to aggregate/agglomerate. The zeta potential of HDPE NPs was roughly between −37 mV and −30 mV, which indicates a high stability of HDPE particles. According to the available literature, zeta potential values ranging between −30 mV to +30 mV are considered optimal for ensuring good stabilisation of dispersion. A high positive or negative zeta potential indicates strong physical stability of the dispersion, as electrostatic repulsion between individual particles reduces the likelihood of aggregation/agglomeration and prevents an increase in particle size [32,33]. In our opinion, a negative zeta potential of the HDPE NPs reflects the medium (e.g., pH, ionic strength, surfactants), since HDPE is a non-polar, hydrophobic polymer that, in its pure form, lacks polar functional groups and surface charges [34]. It should be noted that HDPE NPs were dispersed in DMEM medium with FBS, in EMEM medium with FBS or in PBS. The interaction of nanoparticles with these dynamic environments can modify the initial particle surface. DMEM and EMEM media can induce a negative zeta potential in nanoparticles due to numerous negatively charged components, such as amino acids and ions, which adsorb to the nanoparticle surface, creating a negative surface charge [35]. Specific components in these media, such as bicarbonate buffers, can affect pH, which, in turn, influences the protonation state of a nanoparticle’s surface, altering its overall charge [36]. Moreover, FBS can induce a negative zeta potential on nanoparticles primarily by forming a protein corona on their surface. The proteins in FBS, such as bovine serum albumin, have a negative charge at physiological pH, and their adsorption to the nanoparticle surface makes the overall surface charge negative, which is reflected in the zeta potential [37]. In contrast, commercial citAgNPs were spherical and uniform in size, with a negative surface potential ranging from −37 mV to −28 mV. This negative zeta potential of citAgNPs results mainly from the negatively charged citrate molecules bound to their surface, which create electrostatic repulsion between the particles and prevent them from aggregating/agglomeration and clumping together [38,39,40].

Interestingly, despite the relatively high negative zeta potential of individual NPs, HDPE NPs and AgNPcit dispersions tended to aggregate/agglomerate, resulting in a slight decrease in the number of NPs per millilitre. It is worth noting that the observed changes were not statistically significant, but they did indicate a clear trend. This reduction in concentration corresponded to a tendency of gradual increase in hydrodynamic diameter, consistent with particle aggregation/agglomeration. Nanoparticle aggregation and agglomeration are highly complex processes, ruled by thermodynamics (Van der Waals and/or Brownian interactions). They can occur despite a high positive or negative zeta potential due to other factors such as a high concentration of positive ions, a reduction in surface energy, insufficient surface charge to overcome attractive forces, electrostatic interactions between nanoparticles, the composition of the environment in which the nanoparticles are suspended, the concentration of ions, the pH level, temperature and so on [41,42,43].

Indeed, the hydrodynamic diameter of HDPE NPs and AgNPcit, obtained from NTA and DLS, was larger than expected from SEM. This is a well-known phenomenon, and the difference is attributed to the specificity of the methods and tendency of DLS to skew distributions towards larger particle size in the presence of aggregates/agglomerates [44]. Additionally, the hydrodynamic diameter accounts for the size of the nanoparticle along with protein corona, and solvent molecules or ions that form a layer known as the ‘hydrated layer’. This layer significantly increases the effective size of the nanoparticle in solution compared to its dry state observed in SEM. In solution, NPs also tend to aggregate/agglomerate or form clusters due to the electrostatic interactions, van der Waals forces, or steric hindrance. These aggregates/agglomerates contribute to an increase in the effective size of the nanoparticles, resulting in a larger hydrodynamic diameter compared to individual dispersed particles [45,46].

This was further confirmed by the relatively high PDI (0.7 to 0.9 after 24 h), which suggested diverse NPs’ size distribution. The PDI value tended to increase over time, further confirming processes such as aggregation/agglomeration or the adsorption of biomolecules onto the particle surface, which led to greater heterogeneity in particle size.

The aggregation/agglomeration process did not affect HDPE NPs and AgNPcit uptake, as the NPs were eagerly taken up by HT29MTX cells in a concentration- and time-dependent manner, with significance reaching 6 h and 24 h after NPs’ addition for the highest concentration (0.01 μg/mL). HT29MTX cells also eagerly took up AgNPcit, with significance reached as soon as 2 h after NPs’ addition. In contrast, Caco-2 cells were less prone to take up the NPs, and significant uptake was observed only 24 h after NPs’ addition of HDPE NPs or AgNPcit. Interestingly, HDPE in a concentration as low as 0.01 μg/mL completely inhibited uptake of AgNPcit despite its much higher concentration (15 μg/mL). Similarly, AgNPcit inhibited the uptake of HDPE NPs (Figure 2). This result points to the similarity of mechanisms involved in the uptake of HDPE NPs and AgNPcit. A mutual inhibition of the uptake was already observed by us for 30 nm and 100 nm PSNPs [23]. In line, we have previously reported that the AgNPs inhibit the uptake of amyloid beta in mouse BV-2 microglia cells, likely due to utilising the same uptake mechanisms for cell entrance [47]. Mutual inhibition of the NPs’ uptake when compared to the sum of individual uptakes was also observed by us in the case of the treatment of HepG2 cells with AgNPs combined with other metal/metal oxide NPs [48]. We proposed that all these NPs share a similar mechanism of entry, likely associated with scavenger receptors [23,49]. The mechanisms of nanoparticle penetration into cells, in general, and nanoplastic particles, in particular, have been studied for many years. Therefore, there are many excellent review papers on this regard, to mention a few [50,51,52,53]. In general, these papers reported that, depending on the cell type, nanoparticles can enter cells via endocytosis, which can be classified into clathrin-mediated endocytosis, caveolin-mediated endocytosis, clathrin/caveolae-independent endocytosis, phagocytosis and macropinocytosis. Recently, we conducted a detailed mechanistic study on polystyrene nanoparticle uptake in Caco-2 and HT29 cells [23]. This study revealed that although the general mechanisms of entry of nanoplastic particles into the cell seem to be the same as other nanoparticles (e.g., endocytosis), there are differences between particular cell lines (and likely cell types) regarding the specific ways of entry, depending on the expression of membrane receptors or other proteins (e.g., caveolin).

In contrast to the usually reported low toxicity of the model plastic NPs, HDPE NPs were toxic to HT29MTX and Caco-2 cells growing alone; moreover, the cells varied in their susceptibility. Caco-2 cells were more vulnerable to the adverse effects of HDPE NPs. This result is in agreement with our recent study on the uptake of PS NPs, in which Caco-2 cells were also more susceptible than HT29MTX cells to the toxic effects of PS NPs, despite much lower NP uptake [23]. The Caco-2 cells were also more susceptible to the toxic effects of AgNPcit and to its combination with HDPE NPs. However, in line with the mutual inhibition of uptake, the toxic effect of the AgNPcit and HDPE NPs mixture was much smaller than expected from the sum of the individual toxicities of the NPs. This result is in agreement with our previous report that treatment of HepG2 cells with AgNPs combined with less toxic metal NPs (e.g., gold NPs) resulted in lower toxicity than expected from the sum of individual toxicities of each NPs (subtractive effect) [48].

The lower sensitivity of HT29MTX cells than Caco-2 cells to the toxic action of NPs was also observed by other authors. Araujo et al. [54] reported lower sensitivity of HT29MTX cells than Caco-2 cells when exposed to PLGA NPs, solid lipid NPs, and porous silica NPs. These authors attributed the lower sensitivity of HT29MTX cells to the protective role of mucus production, as the NPs were coated with mucus-adhesive chitosan. Removal of the chitosan coating decreased cells’ viability. In line, Arai et al. [55] reported a protective role of mucus in the uptake of Apple-derived NPs, as the removal of mucus by N-acetylcysteine significantly increased the NPs’ uptake. In general, the mucus layer acts as a barrier to NPs’ uptake and translocation [56]. However, this is true only for positively charged NPs. A membrane-bound and secreted mucins, forming a mucus layer are heavily glycosylated glycoproteins that comprise many negatively charged residues, such as carbonyl groups, sialic acid, or sulphonic acid [57,58], that attract and immobilise positively charged, but not the negatively charged particles [59]. Since AgNPcit and HDPE NPs used in this study were negatively charged, the mucus layer does not prevent their uptake, which is apparent from our results, as mucus-producing HT29TTX cells took up HDPE NPs more readily than Caco-2 cells. The difference in the NPs’ uptake between Caco-2 and HT29MTX cells was previously reported by us for PS NPs of different sizes and was attributed to the difference in endocytosis mechanisms [23].

The higher toxicity of HDPE NPs and AgNPcit to Caco-2 cells compared to HT29MTX cells was reflected in the induction of apoptosis. In monoculture, Caco-2 cells were more susceptible than HT29MTX cells to HDPE NPs- and AgNPcit-induced apoptosis. The early apoptotic stage was observed in Caco-2 cells as early as 24 h after treatment. After 48 h of treatment, the number of early apoptotic cells decreased, while the number of late apoptotic cells increased, especially with AgNPcit and its mixture with HDPE NPs. In contrast, no statistically significant signs of apoptosis were observed in HT29MTX cells. A similar picture appeared in the 3D scheme. Our findings are consistent with those of Najahi et al. [60], who demonstrated that exposure to PET and PE microplastics induced a significant increase in apoptosis, highlighting their toxic effects on intestinal epithelial Caco-2 cells. Furthermore, Ding et al. [61] reported that polystyrene triggers apoptosis in Caco-2 cells. Plastic particles, especially microplastics and nanoplastics, are hypothesised to induce apoptosis because of several mechanisms observed in experimental studies. These mechanisms may involve mitochondrial dysfunction, membrane disruption, inflammation, genotoxicity and/or oxidative stress [62,63,64].

To identify the factors responsible for the induction of apoptosis by HDPE NPs and AgNPcit, we measured the level of oxidative stress. In both cell lines, HDPE NPs, AgNPcit, and their mixture induced oxidative stress as revealed by the DCFDA assay. In contrast, in both cell lines, the DHR probe revealed induction of oxidative stress only with AgNPcit and the HDPE NPs mixture. Both probes have been routinely used in in vitro studies and, due to their limited specificity, are claimed to measure general oxidative stress rather than the specific production of particular oxidant species [65]; however, our results indicate that both probes might respond to different oxidants or exhibit different sensitivities. The oxidative stress induced by the mixture of AgNPcit and HDPE NPs was much smaller than that expected from the sum of the effects of individual NPs, which is in line with the mutual inhibition of the uptake of AgNPcit and HDPE NPs in the mixture. The oxidative stress and its consequences, such as oxidative damage to DNA, lipids, or proteins, or disturbance in cellular signalling, are commonly accepted causes of NPs’ toxicity [66], including AgNPs and nanoplastic (for review see [67,68,69,70]), thus it seems a likely cause of the HDPE NPs’ toxicity. In the 3D triple-culture, regardless of the Caco-2/HT29MTX cell ratio, no oxidative stress was observed with HDPE NPs, AgNPcit, or their mixture.

We also reported different responses of cells growing in 2D monoculture to HDPE NPs, AgNPcit, or their mixture, compared with those growing in 3D triple-culture, in terms of apoptosis induction as an endpoint. Whether this is a general rule or just a phenomenon observed for HDPE NPs and AgNPcit needs further confirmation. Further investigations are also necessary to explain the background of this difference. However, at least three potential explanations come to mind: (1) HT29MTX cells secreted mucus may prevent nanoparticle uptake and protect Caco-2 cells from their toxic; (2) HT29MTX cells and Caco-2 cells compete for NPs’ uptake, and the less sensitive cell line (in our case HT29MTX cells) took up more NPs and protect Caco-2 cell line from their toxic action; (3) presence of Raji cell in 3D triple-culture induces differentiation of Caco-2 cells to the microfold cell (M cells) that differ in susceptibility for undifferentiated Caco-2 cells. The role of the mucus layer in preventing NPs’ toxicity was discussed above, and it seems that this is not the case for negatively charged NPs. Since the majority of mammalian cells share the general NPs’ uptake mechanisms [70], it seems reasonable that cells in coculture will compete for the NPs. Although the experimental design of this project did not allow for distinguishing the NPs’ uptake between single cell lines growing in coculture, in a separate set of experiments, we used fluorescently labelled polystyrene NPs. Our preliminary results suggest that HT29MTX cells indeed took up more NPs than Caco-2 cells when grown in coculture (to be published elsewhere). Finally, it is well established that Caco-2 cells growing in the presence of immune cells (e.g., Raji, THP-derived, or primary macrophages) partially differentiate into functional M cells [71], which may affect their susceptibility. Differentiated Caco-2 cells are usually less susceptible to different stressors, such as oxidative stress [72] or NPs [73], than undifferentiated ones.

## 4. Materials and Methods

### 4.1. Nanoparticles

#### 4.1.1. Silver Nanoparticles

Citrate-stabilised silver nanoparticles (AgNPcit) of nominal size 20 ± 3 nm were purchased from nanoComposix (San Diego, CA, USA, Cat. No. SKU: AGCB20-1M). The photographs of AgNPcit are available from the manufacturer’s website (https://nanocomposix.com/products/20-nm-silver-nanospheres?variant=41238517874777 (accessed on 26 November 2025)).

#### 4.1.2. HDPE Nanoparticles

**Figure 10:**
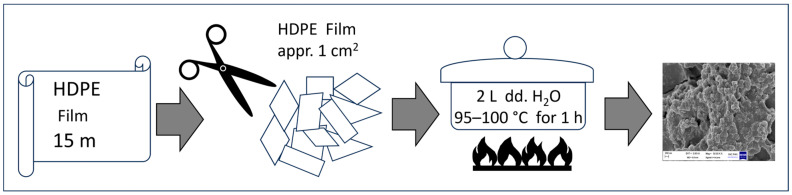
Diagram illustrating the preparation of high-density polyethylene (HDPE) nanoparticles.

High-density polyethylene (HDPE) commercial perforated film used to produce rice cooking bags was purchased from the manufacturer of plastic packaging, PLASTEXIM (Tychy, Poland, January 2022). The procedure for isolating HDPE particles was as follows: a 15-m-long film (equivalent to approximately 100 bags used for cooking rice or groats was cut into smaller fragments measuring about 1 cm^2^ each using steel scissors (Figure 10). The film fragments were then placed in a glass vial containing 2 L of deionised water and heated to 95–100 °C for 1 h using a magnetic stirrer with a heating function. After this period, the film was removed from the glass vial, and the remaining solution was evaporated to a volume of about 10 mL to concentrate the sample. The resulting solution was filtered through a syringe filter with 0.22 µm pores.

### 4.2. Nanoparticle Characterisation

#### 4.2.1. High-Resolution Scanning Electron Microscopy (HR-SEM)

The morphology of the samples was investigated using high-resolution scanning electron microscopy (Carl Zeiss “ULTRA plus”, Oberkochen, Germany, Ultra-High-Resolution Imaging, Jena, Germany). The isolated plastic particles (HDPE) in deionised water (100 µL) were carefully dropped into aluminium specimen stubs (12.5 mm diam., short pin) and sprayed with a thin layer of carbon using a vacuum evaporator (JEE-4X, JEOL, Tokyo, Japan) to ensure conductivity and protect the sample from heat destruction.

#### 4.2.2. Nanoparticle Tracking Analysis (NTA)

Nanoparticle tracking analysis (NTA) was performed using a NanoSight LM20 (NanoSight, Amesbury, UK) equipped with a 640 nm laser and a sample chamber. 1 mL of particle solution in PBS or exposure media (DMEM + 10% FBS for HT29MTX cells and EMEM + 10% FBS for Caco-2 cells) was injected into the sample chamber. All measurements were performed at room temperature. The hydrodynamic size distribution of the microplastic samples was analysed using the NTA 2.0 Build 127 software. Tracking Analysis (NTA) was used to count the concentration of particles. Results include over 200 tracked particles. To examine the effect of time on nanoparticle hydrodynamic diameter and concentration, the nanoparticles were dispersed in PBS or in exposure media containing serum and stored at 37 °C. Aggregation/agglomeration was measured at 0, 0.5, 2, 6, and 24 h.

#### 4.2.3. Dynamic Light Scattering (DLS)

The hydrodynamic diameter, zeta-potential and polydispersity index of the plastic samples were measured at 25 °C in a DTS 1067 capillary cell by using dynamic light scattering (DLS, ZetasizerNanoZS, Malvern, UK). For measurements, the nanoparticle solution in PBS or exposure media (DMEM + 10% FBS for HT29MTX cells and EMEM + 10% FBS for Caco-2 cells) was diluted 1:8 in deionised water and measured in triplicate with 20 sub runs. Zeta potentials were calculated using the Smoluchowski limit for the Henry equation with a setting calculated for practical use (*f*(*ka*) = 1.5). The polydispersity index (PDI) was obtained from the autocorrelation function using a default filter factor of 50%, and default lower and upper thresholds of 0.05 and 0.01, respectively. To examine the effect of increased time on the hydrodynamic diameter, zeta potential, and polydispersity index, the nanoparticles were dispersed in PBS or in exposure media containing serum and stored at 37 °C. Aggregation/agglomeration was measured at 0, 0.5, 2, 6, and 24 h.

#### 4.2.4. Endotoxins

Contamination of nanoparticles with bacterial pyrogens was estimated by assessing the endotoxin level in nanoparticle stock solutions by Limulus amebocyte lysate (Pyro-Gene rFC purchased from Lonza (Walkersville, MD, USA). The PyroGenerFC test utilises recombinant Factor C (rFC), in combination with a fluorogenic substrate. In contrast to the classic LAL assay, rFC does not recognise bacterial glucans that may also be present in nanoparticle solutions. Each nanoparticle solution was tested at a concentration of 100 ng/mL. All procedures were performed according to the manufacturer’s instructions. Briefly, the lyophilised E. coli O55:B5 endotoxin supplied with the kit was reconstituted with the volume of LAL Reagent Water indicated on the vial to yield a 20 EU/mL stock solution. The vial was shaken at high speed for 15 min to reconstitute completely. Standards of the following concentrations were prepared by diluting the stock solution with LAL Reagent Water: 5 EU/mL, 0.5 EU/mL, 0.05 EU/mL, 0.005 EU/mL, and 0.001 EU/mL. Next, the 96-well plate was preincubated in the plate reader at 37 °C for 10 min, and then 100 μL of the test solution or endotoxin standard (in triplicate) was added to the wells. During preincubation, the working reagent was prepared by mixing fluorogenic substrate, assay buffer, and rFC enzyme solution in a 5:4:1 ratio, respectively. 100 μL of working reagent was added to each well. The fluorescence of the solution was measured at excitation/emission wavelengths of 380/440 nm using an Infinite200 Pro plate reader (TECAN, Männedorf, Switzerland). The endotoxin concentration in solutions was determined using a previously established standard curve over the concentration range of 0.5–5.0 EU/mL of E. coli 055 endotoxin. Two independent experiments were done for each nanoparticle solution. Results are presented as a mean and range.

### 4.3. Cell Culture

Human colorectal adenocarcinomas: Caco-2 (ATCC HTB-37™), mucus-secreting, goblet-like HT29MTX (ATCC HTB-38™), and Burkitt lymphoma lymphoblasts (B lymphocyte) (ATCC CCL-86) were obtained from the American Type Culture Collection (ATCC). In monoculture (2D model), Caco-2 cells were cultured in Eagle’s Minimum Essential Medium (EMEM, Biological Industries, Beit HaEmek, Israel) supplemented with 20% foetal bovine serum (FBS, Biological Industries, Israel), whereas HT29MTX cells were cultured in DMEM high glucose medium (Biological Industries, Israel) supplemented with 10% foetal bovine serum (FBS, Biological Industries, Israel). Cells were maintained in a humidified incubator at 37 °C with 5% CO_2_. For subculturing, cells were harvested when they reached approximately 80% confluence.

In a 3D model, a triple-culture of Caco-2, HT29MTX, and Raji cells was cultured on a 3D plate Corning^®^ HTS Transwell^®^-24 well permeable supports (Sigma Aldrich/CLS3398-2EA) in a complete cell medium DMEM (Gibco, Waltham, MA, USA), containing 5% foetal bovine serum (Gibco). 3D cell cultures were maintained at 37 °C and 5% CO_2_. The Caco-2:HT29MTX cells were seeded in two ratios, 70:30 to mimic the proportion of epithelial and goblet cells in the large intestine, or 90:10 to mimic the proportion of epithelial and goblet cells in the small intestine. Subsequently, Raji cells were added to the chamber in the amount corresponding to the total number of cells in the Caco-2/HT29MTX mixture. For better discrimination of cells in the 3D model, in some experiments, Caco-2 cells were transfected with pmScarlet_C1 vector coding red fluorescent mScarlet protein (Ex = 569 nm, Em = 594 nm).pmScarlet_C1 vector was a gift from DrGadella (Addgene plasmid #85042; http://n2t.net/addgene:85042 (accessed on 12 May 2025); RRID:Addgene_85042) [74].

One day before transfection, the cells were seeded in 24-well plates and allowed to reach 70–90% confluency. The cells were transfected using Lipofectamine™ 3000 (Thermo Fischer Scientific, Waltham, MA, USA), according to the manufacturer’s recommendations (https://assets.thermofisher.com/TFS-Assets/LSG/manuals/lipofectamine3000_protocol.pdf (accessed on 12 May 2025)). Briefly, Lipofectamine™ 3000 Reagent was diluted in Opti-MEM™ Medium 3000 (Thermo Fischer Scientific, Waltham, MA, USA). Next, vector DNA was diluted in Opti-MEM™ Medium, and P3000™ Reagent 3000 (Thermo Fischer Scientific, Waltham, MA, USA) was added and mixed. Diluted DNA was mixed with diluted Lipofectamine™ 3000 (1:1 ratio) and left for 15 min at room temperature. DNA-lipid complex was added to cells and incubated for 2–4 days at 37 °C. Transfection efficiency was quantified under an inverted fluorescent microscope (Nikon Eclipse Ti, Tokyo, Japan). During flow cytometry measurements, HT29MTX cells and Raji cells we discriminated by size. The step-by-step discrimination procedure is illustrated in Supplementary Figure S1.

### 4.4. Nanoparticle Uptake Analysis

Nanoparticle uptake analysis was performed in 2D cultures. Cellular uptake of nanoparticles was evaluated by flow cytometry based on changes in side scatter (SSC). Cells were seeded in 6-well plates at a density of 2 × 10^5^ cells/well and allowed to adhere for 24 h. Cells were then exposed to 1 × 10^−2^ μg/mL, 5 × 10^−3^ μg/mL or 2.5 × 10^−3^ μg/mL (roughly corresponding to 0.001 μg/cm^2^, 0.0006 μg/cm^2^ and 0.0003 μg/cm^2^, respectively) HDPE NPs, AgNPcit (40 μg/mL, corresponding to 4.5 μg/cm^2^) or mixture of HDPEs NPs (1 × 10^−2^ μg/mL) and AgNPcit (40 μg/mL) for 2, 6 and 24 h at 37 °C. After treatment, cells were washed with PBS, harvested by trypsinisation, and centrifuged at 300× *g* for 5 min. The cell pellet was resuspended in 1 mL of phosphate-buffered saline (PBS). Samples were immediately analysed by flow cytometry (BD LSR Fortessa, BD Biosciences, Franklin Lakes, NJ, USA). Untreated cells served as a control. Changes in side scatter (SSC-A) were monitored to quantify nanoparticle uptake. Data from 20,000 events per sample were recorded. Cellular uptake of nanoparticles was evaluated only in 2D cultures, as in 3D cultures granularities (SSC values) of Caco-2 and HT29MTX cells overlap, which makes uptake analysis unreliable.

### 4.5. Neutral Red Uptake (NRU) Assay

Neutral Red uptake (NRU) assay was employed to assess nanoparticle cytotoxicity in the 2D model. Cells were seeded in 96-well plates at a density of 1 × 10^4^ cells/well and allowed to adhere for 24 h at 37 °C. Subsequently, cells were exposed to various concentrations of the HDPE NPs (in the range of 1 × 10^−2^–3.13 × 10^−5^ μg/mL) for 24 or 48 h. In a separate set of experiments, the cells were seeded in 96-well plates and treated with 1 × 10^−2^ μg/mL HDPE NPs (~0.001 μg/cm^2^), 15 μg/mL AgNPcit (~4.5 μg/cm^2^), or a mixture thereof. Following the exposure, the culture medium was aspirated, and cells were washed with phosphate-buffered saline (PBS). A Neutral Red (NR) (Sigma Aldrich, St. Louis, MO, USA) working solution (50 μg/mL in the culture medium) was then added to each well and incubated for 3 h at 37 °C. After incubation, the NR solution was removed, and the cells were washed with PBS to remove unincorporated dye. The incorporated NR was solubilised by adding 150 μL of destain solution (50% ethanol, 1% glacial acetic acid, 49% deionised water) to each well. The plates were agitated on a microplate shaker for 15 min to ensure complete dye extraction. The fluorescence was measured at an Excitation Wavelength of 545 nm and an emission wavelength of 630 nm using an Infinite200 Pro plate reader spectrophotometer (TECAN, Männedorf, Switzerland). Results were expressed as a percentage of the viability of untreated control cells, which were set at 100%.

### 4.6. Apoptosis Detection by Annexin V-FITC/PI Staining in 2D Model and by Annexin V-FITC/Sytox Blue Staining in 3D Model

Apoptosis was quantified using the Annexin V-Fluorescein Isothiocyanate (FITC) Apoptosis Detection Kit, according to the manufacturer’s instructions (Thermo Fisher). For apoptosis assessment in 2D model, cells were seeded in 6-well plates at a density of 2.5 × 10^5^ cells/well and treated with HDPE (concentration of 2.5 × 10^−3^, 5 × 10^−3^, and 1 × 10^−2^ µg/mL, roughly corresponding to 0.00023, 0.0056, and 0.001 μg/cm^2^), 40 μg/mL AgNPcit (~4.5 μg/cm^2^) or combination of HDPE (1 × 10^−2^ µg/mL) and AgNPcit (40 μg/mL) for 24 or 48 h. After treatment, both adherent and floating cells were collected. Cells were centrifuged at 800× *g* for 5 min, and the cell pellet was washed twice with cold PBS and then resuspended in 100 μL of the Binding Buffer at a concentration of 1 × 10^6^ cells/mL. Five microliters of Annexin V-FITC solution and 5 μL of Propidium iodide solution (100 μg/mL) were added to each 100 μL of cell suspension. The cells were gently mixed and incubated in the dark at room temperature for 15 min. Following incubation, 400 μL of 1× Binding Buffer was added to each tube. Samples were then immediately analysed by flow cytometry using a BD LSR Fortessa cytometer (BD Biosciences, Franklin Lakes, NJ, USA). For data analysis, compensation was performed using single-stained controls. Data from 20,000 events per sample were recorded. Staurosporine (1 mM) was used to induce apoptosis (a positive control).

Apoptosis was also quantified in the 3D model. Caco-2pmScarlet and HT29MTX cells were seeded into 24-well 3D plate Corning^®^ HTS Transwell^®^-24 well permeable supports. They grew overnight to reach 1 × 10^5^ cells/well on average (in ratio 70:30 or 90:10 of Caco-2pmScarlet:HT29MTX cells, respectively) and treated with 1 × 10^−2^ µg/mL HDPE (0.05 μg/cm^2^), 1.5 μg/mL AgNPcit (corresponding to 0.79 μg/cm^2^) or combination of HDPE (1 × 10^−2^ µg/mL) and AgNPcit (1.5 μg/mL) for 24 h or 48 h. After treatment, both adherent and floating cells were collected and processed in the same way as for the 2D model. The only difference was the replacement of Propidium iodide with Sytox Blue.

### 4.7. Intracellular Reactive Oxygen Species Measurement by Flow Cytometry

Intracellular reactive oxygen species (ROS) levels were determined using flow cytometry with 1,2,3-dihydrorhodamine (123DHR) or 2′,7′-dichlorodihydrofluorescein diacetate (2′,7′-DCFDA) fluorescent probes. For the 2D model, cells were seeded in 6-well plates at a density of 2 × 10^5^ cells/well and allowed to adhere overnight. Cells were then treated with HDPE NPs (2.5 × 10^−3^, 5 × 10^−3,^ and 1 × 10^−2^ µg/mL, roughly corresponding to 0.00023, 0.0056, and 0.001 μg/cm^2^), 40 μg/mL AgNPcit (~ 4.5 μg/cm^2^), or a combination of HDPE NPs (1 × 10^−2^ µg/mL) and AgNPcit (40 μg/mL) for 2 h. Following treatment, the cells were trypsinised, resuspended in fresh medium containing 2% FCS, and the DHR or 2′,7′-DCFDA solution was added to a final concentration of 5 μM. After 30 min incubation with the dye, fluorescence intensity was measured using a BD LSR Fortessa cytometer (BD Biosciences). The fluorescence intensity of DHR or 2′,7′-DCFDA was measured using the FL1 channel (Ex = 488 nm, Em = 530 nm). An increase in the mean fluorescence intensity (MFI) relative to untreated control cells indicated a rise in the level of intracellular reactive oxygen species (ROS). Hydrogen peroxide (1 mM) was used as a positive control.

To quantify ROS production in the 3D model, Caco-2pmScarlet and HT29MTX cells were seeded into a 24-well 3D plate Corning^®^ HTS Transwell^®^-24 well permeable supports and grew overnight to reach 10^5^ cells/well on average (in a ratio of 70:30 or 90:10 of Caco-2pmScarlet:HT29MTX cells, respectively). The co-culture was then treated with HDPE NPs (0.05 μg/cm^2^), 1.5 μg/mL AgNPcit (corresponding to 0.79 μg/cm^2^), or their combination for 2 h. Following treatment, the cells were collected and processed as for the 2D model.

### 4.8. The Concept of Expected Value

Expected value was calculated as a sum of the Control value, the net effect of Treatment 1, and the net effect of Treatment 2:

Exp = Ctrl + (Treatment 1 − Ctrl) + (Treatment 2 − Ctrl)(1)

The effect of the action of two treatments can be additive (both treatments act independently, the experimental value is similar to the expected one), subtractive (both treatments compete, the experimental value is lower than the expected one), or synergistic (both treatments intensify each other’s effect, the experimental value is higher than the expected one).

### 4.9. Statistical Analysis

Data were processed using Graphpad v. 5.0. Data are shown as the mean of three independent experiments, unless otherwise indicated. The Snedecor F test tested the similarity of variances. One-way or two-way ANOVA followed by Tukey, Dunnett, or Bonferroni post hoc comparison, where applicable. A detailed description of statistical analysis is included in the figure’s legend. Significance level was assumed to be lower than 0.05 (*p* < 0.05).

## 5. Conclusions

HDPE NPs thermally isolated from commercial food-cooking bags and AgNPcit are toxic to Caco-2 and HT29MTX cells growing in monoculture. Both cell lines took up the NPs; however, the HT29MTX cell line was more efficient in the NPs’ uptake. Interestingly, NPs mutually inhibited each other’s uptake, which indicates a similar mechanism of entry. The NPs’ toxicity was attributed to the induction of oxidative stress and associated apoptosis. In line with the mutual inhibition of NPs’ uptake, the effect of both NPs in the mixture was less than expected from individual treatments, suggesting a similar mechanism of action. Interestingly, Caco-2 and HT29MTX cells growing in a 3D triple-culture with Raji cells were less susceptible to the toxic action of HDPE NPs and AgNPcit. This is likely an effect of Raji-induced differentiation of Caco-2 cells and lesser vulnerability of differentiated Caco-2 cells to external stressors.

It should be noted that this study also had one limitation: contamination of the HDPE NPs with bacterial pyrogens and endotoxins. This contamination may affect cellular responses, leading to false negatives or positives and skewing toxicity data. However, while pyrogens and endotoxin contamination must be taken into account in in vivo investigations, in vitro cell cultures seem to be more resistant to this factor. To sum up, despite this limitation, we believe that our results extend the small body of existing research and shed light on the potential toxicity of the combined effects of high-density polyethene nanoparticles, originating from in-home natural degradation processes, in combination with silver nanoparticles.

## Figures and Tables

**Table 1:** Hydrodynamic diameter and concentration of HDPE NPs determined by NTA. Data are presented as Mean ± SD from three independent experiments. Statistical analysis was performed on the raw data. Data from the individual experimental groups were compared over time and across media using a two-way repeated-measures ANOVA followed by a Bonferroni post hoc test.

Time [h]	PBS	HT29MTX Medium (DMEM + 10% FBS)	Caco-2 Medium (EMEM + 10% FBS)
Hydrodynamic diameter [nm]			
0	198.7 ± 23.5	207.0 ± 20.1	209.3 ± 25.4
0.5	205.0 ± 28.1	208.0 ± 25.4	211.0 ± 19.2
2	212.0 ± 32.1	219.0 ± 29.8	221.0 ± 35.6
6	224.0 ± 33.9	232.0 ± 32.0	235.0 ± 33.1
24	239.0 ± 30.4	243.0 ± 29.4	245.0 ± 38.7
Concentration [particles/mL]			
0	5.0 ± 0.5 (×10^9^)	4.8 ± 0.5 (×10^9^)	5.3 ± 0.6 (×10^9^)
0.5	4.5 ± 0.6 (×10^9^)	4.8 ± 0.6 (×10^9^)	5.2 ± 0.5 (×10^9^)
2	4.1 ± 0.6 (×10^9^)	4.2 ± 0.6 (×10^9^)	4.4 ± 0.6 (×10^9^)
6	3.5 ± 0.5 (×10^9^)	3.6 ± 0.5 (×10^9^)	3.7 ± 0.5 (×10^9^)
24	2.9 ± 0.4 (×10^9^)	3.1 ± 0.4 (×10^9^)	3.2 ± 0.5 (×10^9^)

**Table 2:** Hydrodynamic diameter, zeta potential, and polydispersity index of HDPE NPs determined by DLS analysis. Data are presented as Mean ± SD from three independent experiments. Statistical analysis was performed on the raw data. Data from the individual experimental groups were compared over time and across media using a two-way repeated-measures ANOVA followed by a Bonferroni post hoc test.

Time [h]	PBS	HT29MTX Medium (DMEM + 10% FBS)	Caco-2 Medium (EMEM + 10% FBS)
Hydrodynamic diameter [nm]			
0	245.23 ± 1.23	260.66 ± 0.77	265.20 ± 1.32
0.5	249.01 ± 0.92	272.23 ± 1.45	272.45 ± 2.22
2	256.92 ± 3.12	298.13 ± 2.45	301.24 ± 3.42
6	273.06 ± 2.08	315.87 ± 5.73	320.33 ± 3.76
24	299.31 ± 2.98	352.43 ± 4.33	361.82 ± 1.02
Zeta potential [mV]			
0	−37.91 ± 1.55	−35.16 ± 5.21	−35.63 ± 2.03
0.5	−36.04 ± 2.06	−34.23 ± 3.22	−34.98 ± 1.67
2	−35.03 ± 4.12	−32.56 ± 1.37	−31.32 ± 3.21
6	−33.08 ± 0.23	−30.44 ± 2.25	−30.98 ± 1.03
24	−30.92 ± 1.79	−29.83 ± 4.02	−29.80 ± 2.84
Polydispersity index (PDI)			
0	0.42 ± 0.02	0.50 ± 0.03	0.50 ± 0.03
0.5	0.45 ± 0.03	0.50 ± 0.03	0.50 ± 0.04
2	0.52 ± 0.04	0.58 ± 0.05	0.57 ± 0.05
6	0.62 ± 0.05	0.68 ± 0.06	0.70 ± 0.07
24	0.73 ± 0.06	0.82 ± 0.07	0.90 ± 0.09

**Table 3:** Hydrodynamic diameter and concentration of AgNPcit determined by NTA. Data are presented as Mean ± SD from three independent experiments. Statistical analysis was performed on the raw data. Data from the individual experimental groups were compared over time and across media using a two-way repeated-measures ANOVA followed by a Bonferroni post hoc test.

Time [h]	PBS	HT29MTX Medium (DMEM + 10% FBS)	Caco-2 Medium (EMEM + 10% FBS)
Hydrodynamic diameter [nm]			
0	36.2 ± 3.1	34.9 ± 0.4	41.6 ± 3.7
0.5	48.3 ± 3.7	52.6 ± 4.1	42.2 ± 4.3
2	21.8 ± 4.9	56.1 ± 3.9	32.0 ± 4.9
6	59.5 ± 4.1	71.5 ± 4.5	61.1 ± 4.1
24	31.9 ± 3.9	65.7 ± 4.4	48.5 ± 4.7
Concentration [particles/mL]			
0	1.8 ± 1.1 (×10^8^)	4.6 ± 4.9 (×10^8^)	6.6 ± 3.6 (×10^7^)
0.5	2.1 ± 4.8 (×10^8^)	5.1 ± 3.7 (×10^7^)	5.7 ± 2.2 (×10^7^)
2	3.0 ± 3.9 (×10^8^)	4.6 ± 3.8 (×10^7^)	4.9 ± 4.5 (×10^7^)
6	5.6 ± 3.5 (×10^7^)	5.7 ± 4.6 (×10^7^)	7.1 ± 1.6 (×10^7^)
24	3.8 ± 2.1 (×10^7^)	7.3 ± 1.9 (×10^7^)	6.8 ± 4.9 (×10^7^)

**Table 4:** Hydrodynamic diameter, zeta potential, and polydispersity index of AgNPcit determined by DLS analysis. Data are presented as Mean ± SD from three independent experiments. Statistical analysis was performed on the raw data. Data from the individual experimental groups were compared over time and across media using a two-way repeated-measures ANOVA followed by a Bonferroni post hoc test.

Time [h]	PBS	HT29MTX Medium (DMEM + 10% FBS)	Caco-2 Medium (EMEM + 10% FBS)
Hydrodynamic diameter [nm]			
0	28.32 ± 2.94	84.32 ± 3.21	80.24 ± 4.23
0.5	30.24 ± 3.11	99.10 ± 4.09	95.12 ± 2.34
2	34.67 ± 4.91	120.45 ± 2.23	116.23 ± 3.41
6	38.21 ± 4.12	138.73 ± 2.32	134.23 ± 4.34
24	46.12 ± 5.43	163.43 ± 4.37	158.67 ± 3.04
Zeta potential [mV]			
0	−36.7 ± 3.21	−35.0 ± 3.33	−35.0 ± 3.21
0.5	−35.9 ± 3.41	−34.5 ± 3.30	−34.6 ± 2.12
2	−34.8 ± 3.60	−33.2 ± 2.13	−33.8 ± 1.21
6	−31.6 ± 4.21	−32.7 ± 2.14	−32.5 ± 2.45
24	−28.2 ± 3.54	−30.01 ± 4.31	−31.0 ± 4.20
Polydispersity index (PDI)			
0	0.12 ± 0.02	0.22 ± 0.03	0.22 ± 0.02
0.5	0.13 ± 0.02	0.26 ± 0.04	0.25 ± 0.03
2	0.16 ± 0.03	0.28 ± 0.02	0.30 ± 0.04
6	0.18 ± 0.04	0.34 ± 0.05	0.34 ± 0.04
24	0.21 ± 0.05	0.38 ± 0.03	0.35 ± 0.05

## Data Availability

Data is contained within the article or supplementary material. The original contributions presented in this study are included in the article/supplementary material. Further inquiries can be directed to the corresponding author.

## Supplementary Materials

The following supporting information can be downloaded at: https://www.mdpi.com/article/10.3390/molecules31010003/s1, (1) Figure S1: The reverse discrimination procedure to discriminate Caco-2, HT29MTX, and Raji cells. (2) Raw data files.

## Abbreviations

The following abbreviations are used in this manuscript:

AgNPcit	Citrate-stabilised silver nanoparticles
PS NPs	Polystyrene nanoparticles
DLS	Dynamic light scattering
DMEM	Dulbecco’s Modified Eagle Medium
EMEM	Eagle’s Minimum Essential Medium
EU	Endotoxin Unit
FBS	Foetal bovine serum
HDPE	High-density polyethylene
NPs	Nanoparticles
NRU	Neutral Red assay
NTA	Nanoparticle Tracking Analysis
PBS	Phosphate-buffered saline
PDI	Polydispersity index
SEM	Scanning electron microscopy
SSC	Side scatter
