# Supplementary material for: Toxicity of High-Density Polyethylene Nanoparticles in Combination with Silver Nanoparticles to Caco-2 and HT29MTX Cells Growing in 2D or 3D Culture
Source: Molecules. 2025 Dec 19;31(1):3. doi: 10.3390/molecules31010003 (PMC12787226; doi:10.3390/molecules31010003)
Supplement: Supplementary file 1 [file molecules-31-00003-s001.zip › Suppl Fig 1 Caco-2, HT29MTX discrimination.pdf]

A B

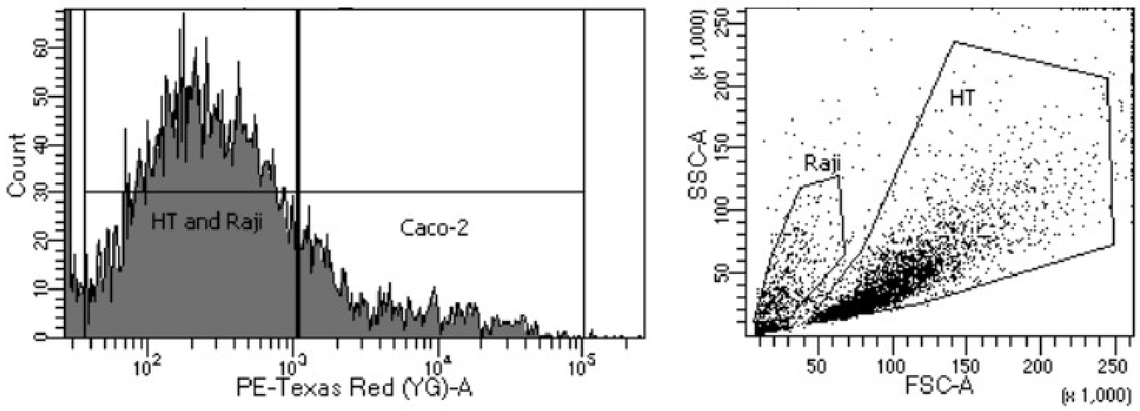

Supplementary Figure S1. The reverse discrimination procedure to discriminate Caco-2, HT29MTX, and Raji cells.

First, pmScarlet-transfected Caco-2 cells (Caco-2pmScarlet) were distinguished based on red fluorescence (the FL2 channel) (A), and then HT29MTX and Raji cells were discriminated based on size and granularity (SSC-A, FSC-A) (B).
